# Amalgams

**Published:** 1875-07

**Authors:** G. A. Bowman


### ARTICLE VII.

### *Amalgams.*

Mr. Editor:—

Among the many and very trying tests recently made of the virtues of the different amalgams in the market, I have not seen the amalgam made by the Chicago Refining Company even mentioned; and it is all the more remarkable because having been on the market for two years or more, and possessing as it does every quality to make it a standard plastic filling. I have used it in my practice for two years, and find nothing to desire in it which it does not possess. I have recently made a few simple tests, which justice compels me to announce through the Journal.

AMALGAM KNOWN AS GOLD AND PLATINA ALLOY.

No. 1. Mixed as usual; washed with soda, and afterwards with alcohol; excess of mercury expressed; packed carefully in pivot tooth; temperature of room, 70°

No. 2. Mixed dry and carefully inserted in pivot tooth without washing.

No. 3. Some of the same mixture, but washed with alcohol; packed in pivot tooth; removing the mercury with a few folds of tin foil under a hot instrument.

Each No. as soon as filled was dropped in an open mouthed vessel of purple ink, in which they remained four days. The most careful inspection could not detect the slighest leak. No. 1 was broken and both filling and cavity conclusively record no leak.

These experiments with this amalgam bear no relation to those of Dr. E. A. Bogue, of New York, in point of scientific exactness, but are sufficiently so to demonstrate its value over the majority of similar compounds.

In addition to these amalgam tests, I carefully filled a pivot tooth with Hill's stopping, and one with common gutta percha, and immersed in the same vessel of ink, remaining four days. The Hill's Stopping resisted successfully the ink, while the gutta percha was less fortunate, leaking badly.—G. A. BOWMAN.
*Missouri Dental Journal.*

---

